# Midkine (MDK) in Hepatocellular Carcinoma: More than a Biomarker

**DOI:** 10.3390/cells13020136

**Published:** 2024-01-11

**Authors:** Christiana Christou, Andreas Stylianou, Vasiliki Gkretsi

**Affiliations:** 1 Cancer Metastasis and Adhesion Laboratory, Basic and Translational Cancer Research Center (BTCRC), European University Cyprus, Nicosia 2404, Cyprus; cc222400@students.euc.ac.cy; 2 European University Cyprus Research Centre Ltd., Nicosia 2404, Cyprus; an.stylianou@euc.ac.cy; 3 Department of Life Sciences, School of Sciences, European University Cyprus, Nicosia 2404, Cyprus; 4 Cancer Mechanobiology and Applied Biophysics Laboratory, Basic and Translational Cancer Research Center (BTCRC), European University Cyprus, Nicosia 2404, Cyprus

**Keywords:** metastasis, EMT, AFP, liver cancer, NEGF-2

## Abstract

Midkine (MDK) is a multifunctional secreted protein that can act as a cytokine or growth factor regulating multiple signaling pathways and being implicated in fundamental cellular processes, such as survival, proliferation, and migration. Although its expression in normal adult tissues is barely detectable, MDK serum levels are found to be elevated in several types of cancer, including hepatocellular carcinoma (HCC). In this review, we summarize the findings of recent studies on the role of MDK in HCC diagnosis and progression. Overall, studies show that MDK is a powerful biomarker for HCC early diagnosis, as it can differentiate not only between HCC patients and normal individuals but also between HCC patients and patients with other liver pathologies. It is correlated with high recurrence rates and was shown to be valuable for the diagnosis of early-stage HCC, even in patients negative for α-fetoprotein (AFP), the most commonly used biomarker for HCC diagnosis. A comparison with AFP reveals that MDK is inferior to AFP with regard to specificity but significantly superior with regard to sensitivity, which further indicates the need for using both biomarkers for more effective HCC diagnosis.

## 1. Introduction

### 1.1. Hepatocellular Carcinoma

Cancer, characterized by uncontrolled cell division and proliferation [1], is one of the most lethal diseases and comprises an important global health challenge. Hepatocellular carcinoma (HCC), in particular, is one of the two main types of primary liver cancer, accounting for 70% of primary liver cancer cases, and in 2020 was ranked sixth among the most frequently diagnosed cancer types and was the third leading cause of cancer-related mortality worldwide [2,3]. In fact, recent data from the United States of America show that, in 2023, 42,210 individuals were estimated to be diagnosed with HCC, and that HCC was estimated to account for 4% of cancer-related mortality in females and 6% in males [4]. Globally, liver cancer was among the top three causes of cancer-related mortality in 46 countries and among the top five causes of cancer-related death in 90 countries. Interestingly, the number of new cases of per year is predicted to increase by 55.0% between 2020 and 2040 [3].

Risk factors that can lead to HCC can be either behavioral, as a result of excessive alcohol consumption and poor diet habits, or viral, due to hepatitis virus infection [5]. In fact, men are more likely to be diagnosed with HCC than women, and age seems to also play a role, with men over 70 having a higher chance of developing the disease [6]. Thus, HCC usually develops in patients with a history of liver cirrhosis due to chronic alcohol consumption or non-alcoholic fatty liver disease (NAFLD) [7]. Also, in approximately half of HCC cases, patients have a hepatitis B virus (HBV) infection [8], while hepatitis C virus (HCV) infection, diabetes, and obesity-related non-alcoholic steatohepatitis (NASH) are other risk factors that contribute to HCC initiation [5,6]. Hence, in most cases, inflammation as a result of a chronic liver disease seems to be triggering HCC development and progression [6].

As with most cancer types, survival rates are significantly better in cases where HCC is diagnosed at an early stage. More specifically, the average survival time of greater than 60 months with early diagnosis drops to less than 15 months with diagnosis at an advanced stage [9]. Thus, the identification of biomarkers for early detection is imperative.

### 1.2. Current Biomarkers Used for HCC Diagnosis and Progression

To date, the most commonly used biomarker for HCC detection is alpha-fetoprotein (AFP). AFP is highly expressed during embryonic development but is not expressed postnatally, with the exception of benign or malignant conditions of the liver, including HCC. Although widely used for HCC detection, its specificity is undermined by its non-specific elevation in non-HCC conditions, such as acute and chronic hepatitis, intrahepatic cholangiocarcinoma, and chronic HCV.

Hence, several other molecules have been suggested as potent biomarkers for HCC diagnosis, as they are found to be dramatically elevated in HCC, including Dickkopf-1 (DKK-1), a secreted glycoprotein that antagonizes Wnt signaling, Golgi protein 73, a transmembrane glycoprotein of the Golgi complex, Glypican-3 (GPC3), a heparan sulphate proteoglycan, osteopontin (OPN), an integrin-binding glycophosphoprotein, des-gamma-carboxy prothrombin (DCP), an abnormal prothrombin with impaired clotting function, and the enzyme gamma-glutamyl transferase (GGT), which is normally secreted by Kupffer cells and whose activity is greatly altered in HCC [10,11].

Apart from serum proteins, several studies have shown the significance of circulating RNAs in HCC diagnosis. Regarding mRNAs, the detection of AFP and GGT mRNA level has been suggested as a potential HCC biomarker, along with that of Toll-like receptor (TLR) mRNAs, which are membrane glycoproteins that serve as crucial components of the innate immune system. Regarding non-coding RNAs, several microRNAs (miRNAs) have been strongly associated with HCC diagnosis, namely miR-500, miR-21, miR-15b, miR-130b, miR-223, miR-26a, miR-27a, miR-122, miR-192, and miR-801 [10].

Most biomarkers are quantified by enzyme-linked immunosorbent assay (ELISA) from the serum samples of cancer patients. However, apart from traditional ELISA, the field of biosensors in HCC detection is rapidly gaining ground. Biosensors combine the technologies of optical, electrochemical, and mass-signal transduction for detecting HCC biomarkers through simple, rapid, sensitive, and cost-effective applications [12]. A biosensor includes a biorecognition receptor layer, which normally contains immobilized enzymes, antibodies, aptamers, and nucleic acids (such as non-coding RNAs) [13]; a transducer; and an electronic system. The biorecognition receptor layer will come into contact with and bind to the biomarker found in the patients’ serum and this interaction will result in measurable physicochemical changes that can be correlated with the concentration of the biomarker.

Regardless of the methodology used for assessing the biomarkers’ levels, low sensitivity and inadequate specificity have been reported for most biomarkers, with the majority of studies concluding that biomarkers should be used in combination for more accurate HCC diagnosis. Thus, the quest for the discovery of novel biomarkers for HCC detection at early or very early stages is ongoing.

In this review, we summarize the findings of recent studies on the role of midkine (MDK) in HCC diagnosis and progression. A comparison with AFP is also made with regard to specificity and sensitivity.

For the completion of this narrative review, a literature search was performed via the PubMed, Scopus and Science Direct databases using the following keywords: HCC, hepatocellular carcinoma, MDK, midkine, NEGF-2, MK, and AFP. All articles that were not written in English were excluded from this study. For all other articles published from 2005 to 2023, the title and the abstract were carefully evaluated; if relevant to the topic of the review, they were further studied, critically evaluated, and presented.

### 1.3. MDK-Mediated Signaling and Cellular Function

**Figure 1:**
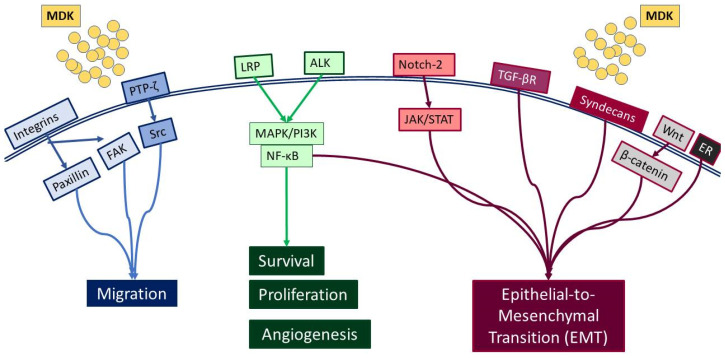
Diagrammatic representation of the receptors interacting with MDK, and the respective signaling pathways that are activated.

MDK is a multifunctional secreted protein that has emerged as a promising biomarker with the potential to aid in early diagnosis and intervention [7]. MDK, also known as neurite growth-promoting factor-2 (NEGF-2), amphiregulin-associated protein (ARAP), mid-gestation and kidney protein, and retinoic acid inducible factor, is a 13 kDa cysteine-rich protein encoded by the *MDK* gene [14], which is located on chromosome 11 [15]. It was originally discovered as a highly expressed gene during mouse embryogenesis [16] and, to date, seven MDK mRNA isoforms have been reported due to alternative splicing and differences in the transcription initiation site [17]. Classified as a heparin-binding protein, MDK can act as a cytokine or growth factor [14,16,18]. It is composed of two domains, each including three antiparallel β-strands and various heparin-binding consensus sites [14], which enable its binding to heparan sulfate and chondroitin sulfate and the formation of a molecular complex with proteoglycans [18]. Importantly, MDK binding to sulfated glycosaminoglycans (GAGs) leads to interactions with several key receptors, including protein-tyrosine phosphatase-ζ (PTP-ζ), syndecans, low-density lipoprotein receptor-related protein (LRP), α4β1 and α6β1 integrins, and neurogenic locus notch homolog protein 2 (Notch-2), while it has also been reported to phosphorylate the cell-matrix adhesion proteins paxillin and focal adhesion kinase (FAK) [19] (Figure 1).

**Figure 2:**
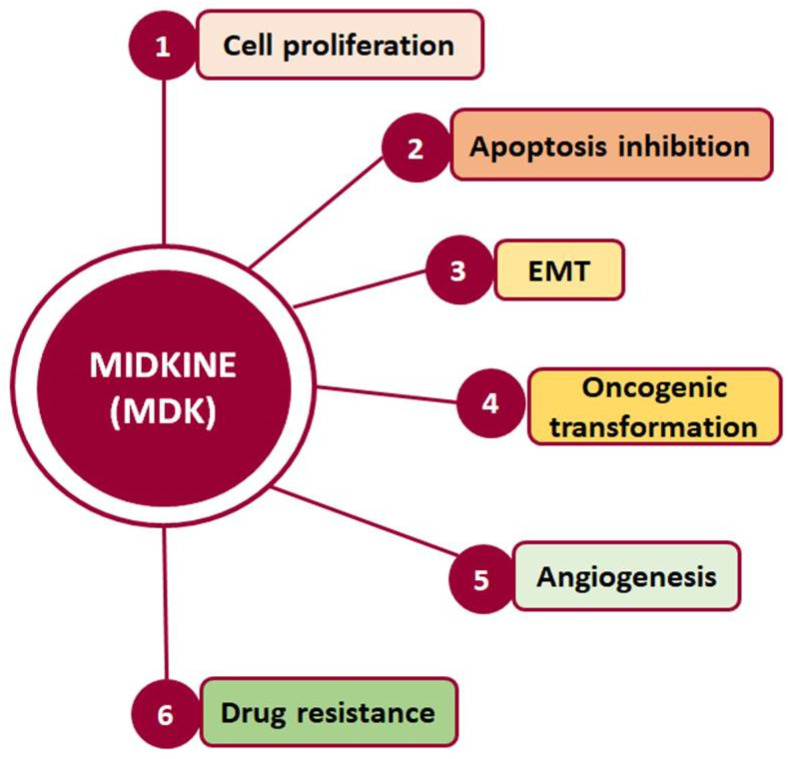
Cellular processes in which MDK is crucially involved and which are related to cancer development and progression.

Such interactions prompt, in turn, the activation of major pro-survival signaling pathways, including Src family kinase, phosphoinositide 3 kinase (PI3K), mitogen-activated protein kinase (MAPK), and nuclear factor kappa B (NF-κΒ), which in turn promote various cellular functions, such as cell proliferation, survival, adhesion, migration, and angiogenesis (Figure 2) [20,21]. Moreover, MDK binding to receptors such as the Notch2, LRP1/integrin, and transforming growth factor beta (TGF-β) receptors activates Janus kinase/signal transducer and activator of transcription 1 (JAK/STAT) and STAT3 [22] as well as MAPK pathways, resulting in epithelial to mesenchymal transition (EMT), a process that defines cancer progression and leads to metastasis [18,20]. It is worth noting that MDK can also induce EMT through interactions with β-catenin via Wnt signaling and the estrogen receptor (ER) [16]. Additionally, its interactions with LRP1, anaplastic lymphoma kinase (ALK), and integrins along with the downstream pathway MAPK/PI3K/Akt lead to increased proliferation and angiogenesis [20,23]. MDK also has the ability to induce cell proliferation by inhibiting apoptosis through the inhibition of caspase-3 and anoikis [23]. Finally, several studies in various cancers highlight MDK as an emerging player in drug resistance [14] (Figure 2).

Hence, through its involvement in the activation of multiple signaling pathways, MDK is implicated in fundamental processes, such as development, reproduction, repair, inflammation, innate immunity, blood pressure control, neurite outgrowth, and angiogenesis [17], while it also promotes cellular activities such as growth, survival, EMT, migration, and invasion, which are crucial in cancer development and progression [24].

### 1.4. MDK Localization, Expression, and Detection

While MDK is considered to be a secreted protein, ultrastructural analysis with immunoelectronic microscopy has also identified the presence of MDK in the nucleolus. Specifically, MDK was found to be localized in the granular component, the dense fibrillar component, and the border between that and the fibrillar center. Moreover, exogenous MDK inhibits apoptosis in the HepG2 HCC cell line and plays an important role in rRNA transcription, ribosome biogenesis, and cell proliferation, confirming a functional role related to its localization [25].

Interestingly, MDK expression in normal adult tissues is undetectable or weak [15]. In fact, the highest expression of MDK is detected in infants, while plasma MDK levels progressively decline with age in healthy children [26]. Interestingly enough, though, it is found to be overexpressed in several types of cancer, including HCC, especially during tumor progression to more advanced stages. Specifically, apart from HCC, MDK expression is found to be elevated in lung cancer [27,28], glioblastoma [29], childhood lymphoblastic leukemia [30], gastric cardia adenocarcinoma [31], prostate cancer [32], head and neck squamous cell carcinoma [33], pancreatic cancer [34], endometrial cancer [35], and bladder cancer [36], and, in most cases, it is also associated with a more metastatic phenotype and a poor prognosis.

This, in conjunction with the fact that MDK is a secreted protein that can be easily detected in the blood, provides an extra advantage, as its levels can be determined through non-invasive blood and urine analyses, and it can therefore be utilized as a biomarker [16].

### 1.5. MDK’s Potential as a Biomarker for HCC Diagnosis and Progression

MDK was therefore proposed as a potential biomarker over a decade ago, as its levels were observed to be elevated in the serum of HCC patients. Indeed, several studies have shown that MDK is upregulated in HCC patients compared with healthy individuals (Table 1).

More specifically, Omran et al. [37] assessed MDK serum levels by ELISA, initially in 104 HCC patients and 92 individuals with non-malignant liver disease, and subsequently in 80 HCC patients and 42 patients with liver cirrhosis. They found elevated MDK levels in HCC patients compared with cirrhotic patients, suggesting MDK’s potential as a distinctive marker in HCC diagnosis and its differentiation from cirrhosis. Along the same line, Malov et al. [38] evaluated MDK serum levels by ELISA in 55 patients with HCV-related liver cirrhosis with concurrent HCC and 55 patients with chronic HCV-related liver cirrhosis without HCC. They also found that MDK had high sensitivity in diagnosing HCC, even among cirrhotic patients, making it a promising biomarker for HCC diagnosis. Moreover, Haque et al. [39] examined serum MDK levels in 30 HCC patients and 30 healthy controls and concluded that patients with chronic HCV-induced HCC had higher serum levels of MDK compared with the control group. They also showed that a combination of MDK and AFP improved the sensitivity of HCC diagnosis and predicted HCC progression. In another study by Darmadi et al. [40], a correlation was sought between MDK levels and tumor size. Indeed, the findings in 100 patients diagnosed with HCC showed that MDK was higher in tumor sizes exceeding 5 cm compared with those with sizes below 3 cm.

Notably, a recent comprehensive study in HCC using bioinformatics analysis approaches identified 10 hub genes from protein–protein interaction networks, among which MDK was further verified by polymerase chain reaction (PCR) and immunohistochemistry (IHC) and found to be highly expressed in HCC [41].

Interestingly, Sun et al. [23] showed elevated MDK levels in the serum of 341 HCC patients who also had curative partial hepatectomy compared with healthy individuals. More importantly, these were positively correlated with increased recurrence rate compared with normal MDK levels. Moreover, they analyzed MDK mRNA expression in nine HCC cell lines (PLC/PRF5/F, Huh7, Hep3B, HepG2, SMMC7721, MHCC97L, and MHCC97H) and two normal liver cell lines (WRL68 and Chang liver) and showed that although MDK mRNA expression varied significantly among the HCC cell lines tested, in all cases it was much higher than that in the normal cell lines used. Moreover, they continued with a series of in vitro and in vivo experiments and reported a negative correlation of differential MDK expression in the cell lines tested with resistance to suspension-induced cell death, known as anoikis. Moreover, PLC/PRF/5 cells lacking MDK via small interfering RNA (siRNA)-mediated silencing exhibited reduced in vitro expression of pro-apoptotic caspase-3 and Bax and a concurrent upregulation of anti-apoptotic B-cell lymphoma 2 (Bcl2) and tropomyosin receptor kinase B (TrkB), a known and potent suppressor of anoikis. Moreover, in vivo studies in BALB/c mice injected with PLC/PRF/5 cells infected with lentiviral vector containing the Gauss luciferase gene and short hairpin RNA (shRNA) against MDK (Lv-Gluc-shMDK) showed lower levels of Lv-Gluc in the blood, indicating that MDK inhibits anoikis in vivo, perhaps as a means to protect circulating tumor cells (CTC) from death when found in circulation. Also, mice injected with MDK-knockdown cells had a higher number of tumor foci on the surface of the liver, further verifying previous findings.

**Table 1:** Studies on the expression of MDK in HCC samples and/or HCC cell lines and comparison with AFP.

Reference	Samples	Methodology	Main Findings	Comparison with AFP
[37]	- Blood serum samples from 104 HCC patients and 92 with non-malignant liver diseases. - Blood serum samples from 80 HCC patients and 42 with liver cirrhosis.	ELISA	MDK levels were upregulated in HCC patients compared with liver cirrhosis patients.	N/A
[38]	- Blood serum samples from 55 HCC patients with HCV-related cirrhosis and 55 with HCV-related cirrhosis without HCC.	ELISA	MDK had high sensitivity for HCC diagnosis vs. cirrhosis.	N/A
[39]	- Blood serum samples from 30 HCC patients and 30 healthy controls.	ELISA	Increased MDK levels in chronic HCV-induced HCC patients compared with controls.	MDK should be used in combination with AFP.
[40]	- Blood samples from 100 HCC patients.	ELISA	MDK was higher in tumor sizes exceeding 5 cm compared with those with sizes below 3 cm.	N/A
[23]	- Cell lines: PLC/PRF5/Huh7, Hep3B, HepG2, SMMC7721, MHCC97H, MHCC97L, WRL68, and Chang liver. - Blood serum samples and tissue samples from 341 HCC patients and healthy controls.	- Real-time PCR - Anoikis, colony formation, and Matrigel invasion assays - Western blotting - Caspase-3 activity assay - CTC enrichment, enumeration, and characterization - ELISA - TUNEL assay - siRNA silencing - Tumor xenografts	MDK mRNA levels were higher in HCC cell lines compared with normal liver cells. Serum MDK was associated with CTC counts and post-operative recurrence in HCC patients. MDK serum levels were upregulated in HCC patients compared with controls. In vitro and in vivo experiments showed that MDK promotes anoikis.	N/A
[42]	- Blood serum samples from 27 patients with chronic HCV, 18 patients with liver cirrhosis, 29 patients with HCC, and 7 healthy controls.	- Real-time PCR	MDK expression was higher in the HCC group.	No correlation with AFP levels.
[15]	- Blood samples from 40 HCC patients, 30 with liver cirrhosis, and 30 healthy controls.	ELISA	MDK was overexpressed in HCC patients.	MDK was better than AFP in HCC diagnosis, especially in the early stages. No correlation with AFP levels.
[43]	- Blood serum samples from 78 HCV-related HCC patients, 40 with HCV related cirrhosis, 40 with chronic HCV without cirrhosis, and 80 healthy controls.	ELISA	MDK was higher in the HCC group compared with other groups and could distinguish between HCC and non-HCC cases, despite the presence of cirrhosis.	MDK’s sensitivity was superior to AFP’s, although AFP’s specificity was higher. MDK should be used in combination with AFP.
[44]	- HCC cell lines: Bel-7402, Huh-7, HCCLM3, MHCC97H, PLC, HepG2, and Hep3B. - Normal cells: L-O2, Chang. - Tissue samples from 88 HCC patients and 88 healthy controls. - Blood serum samples from 388 HCC patients and 545 healthy controls.	- Western blot - TMA - IHC - ELISA	MDK was overexpressed in HCC cell lines compared with normal liver cells.	MDK was better than AFP as it could detect HCC (small-size or early-stage tumors) even in AFP-negative tumors.
[45]	- Blood serum samples from 89 patients with HCV-related cirrhosis without HCC, 86 with HCV-related cirrhosis with HCC, and 69 healthy controls.	- ELISA	MDK was higher in the HCC group compared with other groups. MDK was higher in patients with multiple focal lesions, lesions >5 cm, and portal vein thrombosis. MDK predicted HCC development in HCV-related cirrhotic patients.	MDK was better than AFP in differentiating HCC patients from individuals with liver cirrhosis.
[46]	- Blood serum samples from 84 patients with HCC who were treated with minimally invasive interventional therapy.	- ELISA	MDK was detected in 95% of patients before treatment and decreased after treatment to 67%. MDK was associated with tumor number and size, vascular invasion, and clinical stage. Patients with positive MDK before intervention were more likely to relapse compared with those without MDK expression. MDK expression was detected in 83% of patients with early-stage AFP-negative HCC.	95% of AFP-negative patients exhibited positive MDK expression.
[47]	- Blood serum samples from 35 HCV patients without cirrhosis, 35 HCV patients with cirrhosis, 35 HCC patients with cirrhosis, and 35 healthy controls.	- ELISA	MDK serum levels were elevated in HCC patients.	No correlation with AFP levels. MDK should be used in combination with AFP.
[48]	- Blood serum samples from 44 HCC patients, 31 with cirrhosis, and 15 healthy controls.	- ELISA	Increased MDK levels in HCC patients compared with cirrhotic patients and controls. No correlation was found between MDK and AFP.	No correlation with AFP levels. 80% of AFP-negative patients were MDK-positive. MDK should be used in combination with AFP.
[49]	- Blood serum samples from 85 archival patients with HCC, 165 patients with active HCC, and 285 patients (144 in remission after HCC treatment and 141 at risk for developing de novo HCC). - 18 tissue samples from patients with adjacent normal liver tissue.	- ELISA - Microarrays - Real-time PCR	MDK was elevated in HCC patients compared with high-risk individuals and increased in HCC tissues compared with normal adjacent tissues.	MDK should be used in combination with AFP.
[50]	- Blood serum samples from 46 HCC patients, 46 cirrhotic patients, and 46 healthy controls.	- ELISA	Increased MDK levels in HCC patients compared with cirrhotic patients and healthy controls.	MDK had better diagnostic performance in diagnosing very early and early HCC compared with AFP. MDK mean value differed in HCC patients negative for AFP compared with HCC patients positive for AFP.
[51]	- 40 HCC patients, 30 HCV patients without HCC, and 30 healthy controls.	- Real-time PCR	Increased MDK levels in HCV and HCC groups compared with controls.	
[52]	- Blood serum samples from 86 HCC patients, 86 with liver cirrhosis, 86 with chronic liver disease without cirrhosis, and 86 healthy controls. - Blood serum samples from 28 HCC patients, 28 with HBV-induced cirrhosis, 28 with chronic HBV without cirrhosis, and 28 with HCV-induced cirrhosis.	- ELISA	MDK was higher in the HCC group. MDK was not associated with HCC etiology, but was associated with BCLC staging and high tumor number. MDK could diagnose NASH-related HCC.	- AFP was better than MDK in distinguishing HCC from non-HCC cases and HCV or HBV-associated HCC from liver cirrhosis. - MDK could distinguish NASH-related HCC from cirrhosis. - MDK was elevated 6 months prior to diagnosis in 67% of patients.

N/A: non-applicable, there was no comparison with AFP in this study.

### 1.6. MDK in Comparison with AFP

As AFP has long been considered the gold-standard biomarker in HCC diagnosis, several studies have assessed MDK expression and serum levels in HCC patients in comparison with AFP (please see Table 1 and Table 2).

Few studies showed no correlation with AFP levels. Specifically, Saad et al. [42] determined MDK mRNA expression using real-time PCR in 29 patients with HCC and compared it with that of 7 healthy individuals, 27 patients who had chronic HCV, and 18 patients who had liver cirrhosis. They found that MDK mRNA expression was higher in HCC patients compared with the other groups. However, no significant correlation was observed between MDK and tumor characteristics (number of nodules, lesion size, and extrahepatic metastases) or AFP levels. In agreement with the above, Shaheen et al. [15], evaluated MDK protein levels as a biomarker in patients with newly diagnosed HCC. Among 100 participants, including 40 HCC patients, 30 liver cirrhosis patients, and 30 controls, MDK was found to be significantly upregulated in HCC patients compared with both liver cirrhosis patients and controls but showed no association with tumor diameter, number of nodules, AFP levels, or Barcelona Clinic liver cancer (BCLC) staging, which is widely used for staging primary liver cancer. However, it is worth noting that MDK showed greater sensitivity compared with AFP in HCC diagnosis, especially in the early stages, highlighting its potential as a novel marker, especially in differentiating HCC from liver cirrhosis.

The majority of studies, however, showed that MDK provides some additional advantages as a biomarker compared with AFP (Table 1). Specifically, in a recent study [43], serum MDK levels were assessed in 238 individuals, including 78 HCC patients with HCV-related HCC, 40 with HCV-related liver cirrhosis, 40 with chronic HCV without liver cirrhosis, and 80 healthy controls. The findings consistently confirmed elevated MDK levels in HCC patients compared with other groups. However, although no association with tumor size was found, and while there was no significant difference in MDK protein expression between the other groups, the authors highlighted that MDK diagnostic accuracy for HCC diagnosis was high. Likewise, MDK exhibited the capacity to successfully distinguish between HCC and non-HCC cases, despite the presence of cirrhosis. MDK alone showed a sensitivity of 88.5%, a specificity of 80.6%, and a total accuracy of 83.2%. In fact, MDK’s sensitivity was superior to AFP’s (74,4%), although AFP’s specificity was higher (84.4%). However, when combined with AFP, the sensitivity was 91%, the specificity was 76.9%, and the overall accuracy was 82.35%. Table 2 summarizes results on MDK sensitivity and specificity in comparison with AFP.

Interestingly, in a recent phase II validation study, Zhu et al. [44] evaluated MDK as a diagnostic biomarker in early-stage HCC for those with negative AFP. Their study also included in vitro work in nine HCC cell lines (Bel-7402, Huh-7, HCCLM3, MHCC97H, PLC, HepG2, and Hep3B, with L-O2 and Chang liver cells serving as controls), as well as evaluation of 88 HCC samples, their corresponding adjacent tissues, and serum samples from 388 HCC cases and 545 controls. MDK expression was evaluated using IHC in tissue microarrays (TMA), ELISA in serum samples, and Western blotting in cell lines. They found elevated MDK protein expression in all HCC cell lines compared with controls, while IHC analysis in the TMA exhibited high MDK expression in the form of diffused cytoplasmic staining in 72% of HCCs compared with normal adjacent liver tissue or cancer-free cirrhotic samples. Although serum MDK levels did not exhibit correlations with tumor aggressiveness indicators, such as poor differentiation, microvascular invasion, larger tumor size, advanced tumor stage, survival, and tumor recurrence, they did correlate with MDK expression in tumor tissues. No association was found between MDK and BCLC staging. Compared with AFP, in AFP-positive patients, MDK and AFP had similar specificities; however, MDK showed superior sensitivity to AFP (86.9% and 51.9%, respectively). On the other hand, in patients with early-stage HCC and negative AFP, MDK had better performance compared with AFP for distinguishing early-stage HCC and small-sized tumors from non-HCC cases, including cirrhosis. More specifically, the sensitivity of MDK in detecting early-stage BCLC was 87.1%, compared with 46.7% for AFP. However, the sensitivity of MDK decreased when detecting the very early stages of HCC (80%) compared with AFP (40%). Remarkably, MDK sensitivity (89.2%) was shown to be independent of serum AFP levels, even in those with AFP-negative HCC. Lastly, the study showed a decrease in MDK levels in 36 patients after tumor resection, followed by an elevation at the time of tumor recurrence, further supporting the notion that MDK could be a potent biomarker for disease progression monitoring as well.

In another study, El-Shayeb et al. [45] tested MDK serum levels in 89 patients with liver cirrhosis without HCC, 86 patients with cirrhotic HCV-induced HCC, and 69 healthy controls. They found serum MDK levels to be increased in HCC patients compared with the other two control groups. Interestingly, however, MDK exhibited higher levels in patients with multiple focal lesions, lesions exceeding 5 cm, and those with portal vein thrombosis, compared with those with single focal lesions, lesions smaller than 5 cm, and those without portal vein thrombosis. Finally, MDK was proven to have superior performance compared with AFP in differentiating HCC patients from individuals with liver cirrhosis. Correspondingly, Zheng et al. [46] evaluated the clinical importance of serum MDK levels both for HCC diagnosis and the monitoring of treatment efficacy in samples from 84 HCC patients undergoing minimally invasive interventional therapy. Results showed a correlation between MDK and several clinical characteristics, such as tumor number and size, vascular invasion, and clinical stage, specifically evident in mid-to-late-stage HCC. Notably, MDK was detected in 95% of patients pre-intervention, a proportion that decreased to 67% post-intervention, while patients exhibiting positive MDK expression before the intervention also showed a higher likelihood of relapse compared with those without MDK expression. Interestingly, 95% of AFP-negative patients exhibited positive MDK expression, emphasizing MDK’s role in improving HCC detection rates. Of significance, MDK expression was detected in 83% of patients with early-stage AFP-negative HCC, leading to the observation that MDK detection is a powerful supplement to AFP detection for the diagnosis of HCC, especially in early-stage and AFP-negative HCC.

Furthermore, in a study by Hodeib et al. [47], the diagnostic utility of serum MDK compared with AFP for the diagnosis of HCC in HCV-related liver cirrhosis was evaluated. The study involved a total of 140 participants, encompassing 35 HCV patients without cirrhosis, 35 with HCV and liver cirrhosis, 35 with HCC on top of liver cirrhosis, and 35 healthy controls, and serum MDK levels were evaluated by ELISA. Their main findings showed that serum MDK levels were elevated in HCC patients compared with the other groups and did not correlate with AFP levels, but that MDK had better sensitivity in diagnosing HCC compared with AFP (98.4% vs. 97%). However, the combination of MDK and AFP had a diagnostic value of 98% for HCC diagnosis.

Corroborating further the idea that a combination of MDK and AFP increases diagnostic capacity for HCC, Mashaly et al. [48] also evaluated serum MDK levels by ELISA in 44 HCC patients, 31 with liver cirrhosis, and 15 healthy controls. They also found increased MDK levels in HCC patients compared with cirrhotic patients and controls. More importantly, 21 out of the 44 HCC patients were AFP-negative, but 17 of these AFP-negative patients were MDK-positive, while no correlation was revealed between MDK and AFP, suggesting the independent nature of MDK’s increase in relation to AFP. Additionally, Saeed et al. [50] included in their study 46 HCC patients, 46 liver cirrhosis patients as the disease control group, and 46 healthy individuals as the healthy control group. Serum MDK levels were higher in HCC patients compared with the liver cirrhosis and control groups. Moreover, MDK achieved high sensitivity (91%) and specificity (90%) scores in detecting HCC compared with non-HCC patients, with an overall accuracy of 91.1%. On the other hand, AFP’s sensitivity was only 56%, while its specificity was the same as for MDK. The overall accuracy of AFP was 71%. Similarly, MDK’s diagnostic performance in detecting very early HCC with lesions smaller than 2 cm scored 94% for sensitivity and 91.3% for specificity, while AFP’s sensitivity was slightly smaller (70%) with a specificity of 86%. Overall, MDK’s diagnostic performance in detecting early HCC with lesions of 2–5 cm showed 96% sensitivity and 82.6% specificity. Again, AFP’s sensitivity was lower (50%), but its specificity was 80%. Lastly, the diagnostic performance of MDK in detecting HCC in patients with negative AFP was 85% sensitivity and 88% specificity, making MDK a good candidate for detecting very early, early, and, in some cases, AFP-negative HCC.

Similarly, Hung et al. [49] included in their study four groups: 18 samples from HCC tissues with adjacent non-cancerous liver tissues, 85 archival HCC patient samples, 165 samples from patients with active HCC, and 285 samples from individuals in remission after HCC treatment or at high risk for HCC development. MDK serum levels were assessed by ELISA, while gene expression in the tissue samples was evaluated with microarrays and real-time PCR. MDK mRNA expression was shown to be elevated in all HCC samples compared with the respective adjacent tissue samples. When measuring serum MDK and AFP levels in the 85 archival patients, they found that 25% had elevated levels of both MDK and AFP, 16% had elevated MDK levels only, 33% had elevated AFP levels only, and 26% had neither elevated MDK nor AFP, suggesting that the combination of MDK and AFP can increase the sensitivity of HCC detection. In order to assess the specificity of serum MDK in HCC detection, they also measured serum MDK levels in 72 patients with newly diagnosed HCC from the follow-up group. Of these, 60% had elevated MDK levels. Moreover, they examined changes in serum MDK levels over time in 165 HCC patients. Among them, disease progressed in 122 patients, 41 had stable disease, and 2 showed a partial response to treatment, suggesting a potential correlation between serum MDK levels and disease activity. More specifically, a positive correlation between disease progression and MDK levels was found in 81.2% of patients, while 18.8% showed a negative correlation. Notably, 108 of the 165 patients died as a result of disease progression, with rapidly rising MDK serum levels observed during their last days.

Finally, Daif et al. [51] evaluated the gene expression of MDK in HCC patients compared with serum AFP levels in 40 HCC patients, 30 HCV patients with no evidence of HCC, and 30 healthy controls. Gene expression levels were assessed using qRT-PCR, and MDK mRNA expression was elevated in the HCC and HCV groups compared with controls. Contrary to the other studies described above, MDK alone showed lower sensitivity (50%) and specificity (68.2%) in detecting HCC compared with AFP (78.6% and 72.7% respectively). However, the combination of MDK and AFP reached a sensitivity of 71.4% and a specificity of 81.8%.

Although most studies clearly demonstrated a strong, indisputable correlation between MDK levels and HCC disease progression as well as certain benefits in terms of early detection, sensitivity, or specificity compared with AFP, there was one study by Vongsuvanh et al. [52] showing elevated MDK levels in HCC compared with other groups (86 HCC, 86 liver cirrhosis, 86 non-cirrhotic chronic liver disease, and 86 controls), but, when compared, AFP was proven to be superior to MDK in distinguishing HCC from non-HCC cases and HCV or HBV-associated HCC from liver cirrhosis. However, MDK could differentiate NASH-related HCC from cirrhosis. In a later phase, however, they recruited 112 individuals, including 28 with HCC, 28 with HBV cirrhosis, 28 with non-cirrhotic chronic HBV, and 28 with HCV cirrhosis. MDK was elevated in 54% of the HCC patients at the time of diagnosis and, remarkably, MDK was elevated 6 months prior to diagnosis in 67% of patients, suggesting MDK’s role in pre-clinical HCC diagnosis.

Last but not least, a comparison of MDK’s specificity and sensitivity with the published specificity and sensitivity of in vitro diagnostic assays such as the GAAD or GALAD score-based models shows that MDK is comparable to both GAAD and GALAD scores. More specifically, the average MDK specificity based on the 11 studies described above (Table 2) is 80.34%, while the GAAD score’s specificity is 90% [53] and the GALAD score’s average specificity is 87.71% [54,55,56]. With regard to sensitivity, the average MDK sensitivity value from the 11 studies described above (Table 2) was 83.77%, compared with 71.8% (for GAAD) [53] and 85.82% (the average for GALAD) [54,55,56]. This further demonstrates that MDK could be used in the clinic in combination with AFP, GAAD, and GALAD scores for more effective HCC diagnosis.

## 2. Conclusions

**Figure 3:**
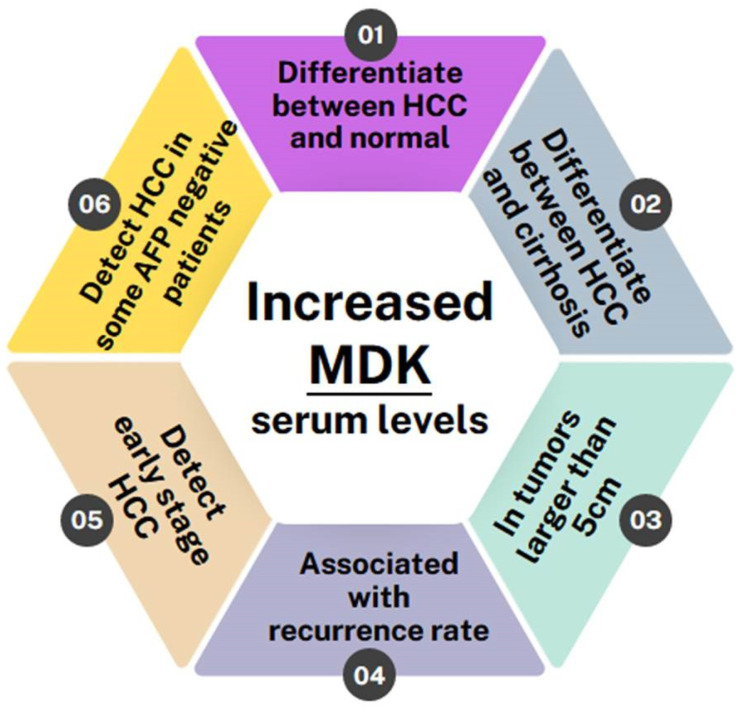
Schematic showing the benefits of MDK as a biomarker in HCC detection.

Overall, all of the studies conducted so far indicate that MDK levels are elevated in HCC patients and can, in fact, differentiate not only between HCC patients and normal individuals but also between HCC patients and patients with other liver pathologies, such as liver cirrhosis, regardless of whether it is HCV/HBV-induced or not [25,26,27,31,33,35]. Furthermore, two studies have shown MDK levels to be correlated with a tumor size greater than 5 cm [28,33] and it is also associated with high recurrence rates [20]. MDK was also shown to monitor disease progression and response to therapy, as it remains elevated in patients with incompletely treated or recurrent HCC while it drops after curative surgery [9]. Most importantly, though, MDK levels can be used to diagnose early-stage HCC and can even diagnose HCC in patients who have tested negative for AFP [11,42,44,45,46] (Figure 3).

However, as there was a report showing some HCC cases with positive AFP and negative MDK [39], MDK should be used in combination with AFP to ensure more effective diagnosis. This is also evident from the analysis of specificity and sensitivity data in Table 2, where it seems that MDK is inferior to AFP with regard to specificity but significantly superior with regard to sensitivity.

## 3. Future Directions

MDK is definitely a molecule of interest, as its use clearly offers certain advantages, such as the ability to diagnose early HCC, the ability to differentiate between HCC patients and normal individuals or patients with other liver pathologies, the ability to predict recurrence, and the ability to monitor disease progression and treatment response [9]. Our analysis of recent literature shows that, in combination with AFP, it can enhance early and more effective HCC diagnosis and is definitely more sensitive than AFP, as it can even diagnose HCC in patients who are AFP-negative. Future research should therefore focus on studying both MDK and AFP in in vitro diagnostic assays such as the GAAD or GALAD score-based models, which could potentially predict HCC even in cases of small nodules. This would be extremely valuable to clinicians in making a safe HCC diagnosis at an early stage.

Moreover, as the biosensor field is rapidly developing, research on the biosensor-aided detection of MDK may pave the way for the development of a system that can combine both MDK and AFP at the same time. One such study recently showed an MDK immunosensor to have high sensitivity, selectivity, stability, and reproducibility [57], which is promising for all future endeavors in that direction.

From the research point of view, although several details are known regarding the molecular mechanism of MDK’s action in general, little is known regarding the specific pathways that are being activated in HCC, as most studies are focusing on its evaluation as a diagnostic biomarker. It is therefore imperative to increase research efforts towards the understanding of MDK’s action in HCC in particular, as well as its evaluation as a potential therapeutic target to treat HCC and/or HCC-related metastasis.

## Figures and Tables

**Table 2:** Studies specifically comparing MDK and AFP in terms of sensitivity and specificity as HCC diagnostic biomarkers. Highlighted in grey are studies showing MDK to be superior to AFP.

Reference	MDK Cutoff Value (ng/mL)	MDK		AFP Cutoff Value	AFP	
		Specificity (%)	Sensitivity (%)		Specificity (%)	Sensitivity (%)
[44]	0.654	86.8	86.9	20 ng/mL	86.8	51.9
[15]	0.387	83.3	92.5	88.5 ng/mL	96.7	40
[52]	0.440	62.2	70.9	24 ng/mL	96.5	43
[47]	0.650	96.2	98.4	80 ng/mL	95	97
[48]	1.683	83.87	81.82	200 ng/mL	96.77	52.27
[37]	1.000	79	76	400 (U/L)	100	29
[38]	0.800	63.6	85.5	20 ng/mL	94.5	45.5
[43]	0.152	80.6	88.5	10.05 ng/mL	84.4	74.4
[45]	5.100	90	100	10 ng/mL	45	78
[51]	1.930	68.2	50	7.55 ng/mL	72.7	78.6
[50]	34.00	90	91	21.5 ng/mL	90	56

## Abbreviations

AFP: alpha-fetoprotein, ALK: anaplastic lymphoma kinase, ARAP: amphiregulin-associated protein, Bax: Bcl2-associated X-protein, Bcl2: b-cell lymphoma 2, BCLC: Barcelona Clinic liver cancer, CTC: circulating tumor cells, DCP: Des-gamma-carboxy prothrombin, DKK-1: Dickkopf-1, ELISA: enzyme-linked immunosorbent assay, EMT: epithelial to mesenchymal transition, ER: estrogen receptor, FAK: focal adhesion kinase, GAG: glycosaminoglycans, GGT: gamma-glutamyl transferase, GPC3: Glypican-3, HBV: hepatitis B virus, HCC: hepatocellular carcinoma, HCV: hepatitis C virus, IHC: immunohistochemistry, JAK: Janus kinase, KLK: kallikrein-related peptidases, LRP1: low-density lipoprotein 1, Lv-Gluc: lentiviral vector containing the gene of Gaussia luciferace, MAPK: mitogen-activated protein kinase, MDK: midkine, miR: micro-RNA, NF-κB: nuclear factor kappa beta, NAFLD: non-alcoholic fatty liver disease, NASH: non-alcoholic steatohepatitis, NEGF-2: neurite growth-promoting factor-2, NOTCH2: neurogenic locus notch homolog protein 2, OPN: osteopontin, PI3K: phosphoinositide 3 kinase, PTP-ζ: protein-tyrosine phosphatase-ζ, shRNA: small hairpin RNA, siRNA: small interfering RNA, STAT: signal transducer and activator of transcription, TGF-β: transforming growth factor beta, TLR: Toll-like receptor, TrkB: tropomyosin receptor kinase B, TMA: tissue microarrays.
